# An Ultra-Rapid Biosensory Point-of-Care (POC) Assay for Prostate-Specific Antigen (PSA) Detection in Human Serum

**DOI:** 10.3390/s18113834

**Published:** 2018-11-08

**Authors:** Sophie Mavrikou, Georgia Moschopoulou, Athanasios Zafeirakis, Konstantina Kalogeropoulou, Georgios Giannakos, Athanasios Skevis, Spyridon Kintzios

**Affiliations:** 1 Laboratory of Cell Technology, Faculty of Biotechnology, Agricultural University of Athens, Iera Odos 75, 11855Athens, Greece; sophie_mav@aua.gr (S.M.); skevis.zhth@gmail.com (A.S.); skin@aua.gr (S.K.); 2 Army Share Fund Hospital of Athens, Monis Petraki 10, 11521 Athens, Greece; athzafeirakis@gmail.com (A.Z.); tankalogero@yahoo.com (K.K.); apathnimts@gmail.com (G.G.)

**Keywords:** bioelectric recognition assay, cellular biosensor, high-throughput, immunoassay, molecular identification through membrane engineering, point-of-care, prostate cancer, prostate-specific antigen

## Abstract

Prostate-specific antigen (PSA) is the established routine screening tool for the detection of early-stage prostate cancer. Given the laboratory-centric nature of the process, the development of a portable, ultra rapid high-throughput system for PSA screening is highly desirable. In this study, an advancedpoint-of-care system for PSA detection in human serum was developed based on a cellular biosensor where the cell membrane was modified by electroinserting a specific antibody against PSA. Thirty nine human serum samples were used for validation of this biosensory system for PSA detection. Samples were analyzed in parallel with a standard immunoradiometric assay (IRMA) and an established electrochemical immunoassay was used for comparison purposes. They were classified in three different PSA concentration ranges (0, <4 and ≥4 ng/mL). Cells membrane-engineered with 0.25 μg/mL anti-PSA antibody demonstrated a statistically lower response against the upper (≥4 ng/mL) PSA concentration range. In addition, the cell-based biosensor performed better than the immunosensor in terms of sensitivity and resolution against positive samples containing <4 ng/mL PSA. In spite of its preliminary, proof-of-concept stage of development, the cell-based biosensor could be used as aninitiative for the development of a fast, low-cost, and high-throughput POC screening system for PSA.

## 1. Introduction

Prostate cancer is one of the four most common cancer types (along with lung, breast, and colorectal cancer) [1,2]. It is characterized by a higher incidence in older age, obesity and white race males, but is also characterized by a low to very low mortality. Detection of the disease at an early stage can lead to very high five-year survival rates (80% or even higher). While digital rectal examination of the prostate gland may fail to detect cancer at an early stage, routine screening in blood for the prostate-specific antigen (PSA), a 28.4 kD serine protease secreted by the epithelial lining of the periurethral glands and the prostatic epithelium, has increased the diagnostic efficiency of prostate cancer from 8% to 18% in less than three decades [3]. However, the reliability of the test has been frequently challenged, particularly in view of its limited usefulness for patients demonstrating a low-to-medium, “gray zone” PSA concentration (4–10 ng/mL). In fact, the prognostic significance of PSA values below 10.0 ng/mL is doubtful [4]. Recent studies suggest an increase in the frequency of PSA testing in order to determine the rate PSA levels elevated prior to diagnosis of prostate cancer (“PSA velocity”) as a more realistic approach in prostate cancer risk stratification [5]. Obviously, scaling up of PSA testing intensity would require the availability of more flexible screening tools, preferably based on a point-of-care (POC) biosensor concept and offering a combination of reliability, speed, low-cost and ease of use.

A number of different electrochemical biosensing approaches hasbeen reported in recent years employing a variety of biorecognition elements for the detection of PSA either in standard solutions or in biological samples. As an analytical concept, electrochemistry offers the advantage of rapid testing, relatively low cost and, quite often, portability. Among the very recent developments in this direction, electrochemical immunoassays stand out as the most favorable working principle. For example, Vural et al. [6] developed a disposable chronoamperometric immunoassay by modifying a graphite electrode with polyaniline (PANI) conjugated with a complex ofa peptide nanotube (PNT) and gold nanoparticles (AuNP) (PANI/AuNP-PNT). A sandwich immunoassay was applied, where PSA was first captured by the composite, followed by attachment of horseradish peroxidase (HRP) conjugated with anti-PSA (HRP-Ab2) antibody. Quantitative changes in electro-catalytic reduction of H_2_O_2_ were directly associated with PSA concentration, with a limit of detection (LOD) of 0.68 ng/mL and a recommended assay time of at least 30 min. Pihikova et al. [7] reported the application of electrochemical impedance spectroscopy (EIS) with a sandwich immunoassay based on a combination of anti-PSA antibody and the *Maackia amurensis* agglutinin (MAA) lectin to glycoprofile captured PSA on the sensing electrode. This approach reduced considerably the non-specific binding and allowed for a very low LOD (100 ag/mL). The assay time was here again 30 min. Significant progress has been reported in the direction of miniaturizing PSA biosensors. Chen et al. [8] fabricated a Field Effect Transistor (FET)-based biosensor employing anti-PSA antibodies immobilized on horizontally aligned carbon nanotubules (CNTs). A LOD of 84 pM PSA was achieved with a total assay time of 135 min. In a more practical sense, paper-based biosensors for PSA screening have been also recently reported. For example, a conductivity PSA paper biosensor was reported by Ji et al. [9] using anti-PSA antibody-bioactivated multiwall carbon nanotubes (LOD = 1.18 ng/mL, assay time = 2 h). Zheng et al. [10] developed an all-around conductive microfluidic paper-based analytical device (μPAD) with cyclodextrin-functionalized gold nanoparticles (CD@AuNPs) immobilized on the paper working electrode via a custom peptide (CEHSSKLQLAK-NH2). When present in the sample, PSA cleaved the peptide, resulting in measurable changes in current flowing through the electrode (LOD = 1 pg/mL, assay time > 40 min). The same peptide-breakage-based approach was used by Yang et al. [11] to develop a fluorescence biosensor. 5-FAM-labeled peptides immobilized on magnetic Fe_3_O_4_@SiO_2_-Au nanocomposites (MNCPs) were specifically recognized and cleaved by PSA, therefore releasing the formerly quenched fluorescence. A LOD of 0.3 pg/mL was achieved with this method (assay time > 1.5 h). Finally, Xu et al. [12] reported the fabrication of a superwettable microchip for PSA immunoassay (among other biomarkers). A nanodendritic gold substrate was electrochemically deposited on a Ti/Au thin film and then modified to achieve a conductive super hydrophobic-superhydrophilic surface. This enabled the drastic reduction of the sample volume, while the diameter of the electrode microwell ranged from 0.5 to 2 mm. Another worthy attempt for developing diagnostic tools for prostate cancer was the Marie Curie Initial Training PROSENSE (www.prosense-itn.eu). In the framework of this project a lot of studies were published by developing impedimetric aptasensorwith LOD lower than 1 ng/mL and 4 aM [13,14], electrochemical immunosensor based on platinum nanoparticles [15] and electrochemical detection of PSA by using DNA aptamer [16]. Tamboli et al. [17] constructedhydrid synthetic receptors that were used in an extended gate field-effect transistors for PSA detection. A LOD of 0.1 pg/mL was achieved.

In the present study, we used the molecular identification through membrane engineering (MIME) and the bioelectric recognition assay (BERA) technology for the development of an innovative point-of-care system for PSA detection in human serum. BERA in combination with MIME technology is a method based on the change of engineered (with specific antibody) cell membrane potential when they interact with the target antigen. This combined approach offers the capability of ultra-rapid detection (3–5 min) and very high sensitivity [18,19,20,21,22]. A set of human serum samples were used for validation of this biosensory system for PSA detection, which were analyzed in parallel with a standard immunoradiometric assay (IRMA) and an established electrochemical immunoassay, that was used for comparison purposes.

## 2. Materials and Methods

### 2.1. Materials

The renal cell line Vero was originally obtained from LGC Standards (Teddington, Middlesex, UK). Dulbecco’s Modified Eagle’s Medium (DMEM), l-alanine-glutamine, fetal bovine serum (FBS), penicillin/streptomycin and trypsine/EDTA were purchased from Biochrom Ltd. (Cambridge, UK). The PSA antibody and all other reagents were ordered from Sigma-Aldrich (Munich, Germany). Thirty nine whole blood samples with PSA values measured by IRMA technique in the range from zero up to 224.4 ng/mL, were collected from an equal number of patients at Army Share Fund Hospital of Athens. Prior to assay, samples were stored at −20 °C for one up to three months. All the screen printed electrodes were provided from DropSens (Llanera, Asturias, Spain).

### 2.2. Manufacture of the Biosensing Element

**Figure 1:**
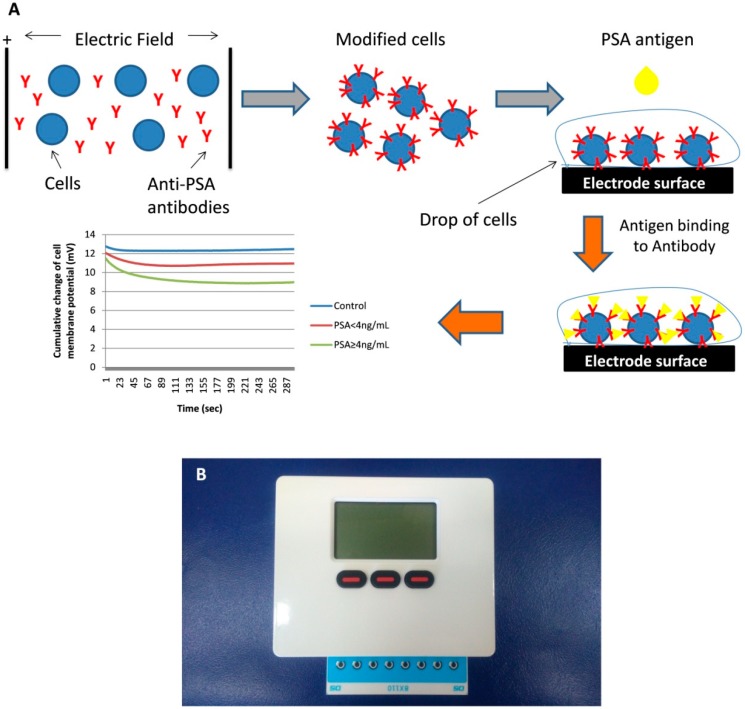
(**A**) Schematic picture of the assay workflow (**B**) Portable biosensor read-out device with a disposable eight-position screen-printed electrode strip.

Vero cell line was cultured in DMEM with 10% FBS, 1% antibiotics (penicillin/streptomycin) and 1% l-alanine-glutamine. Cells were detached from the culture flask with addition of trypsin/EDTA for 5 min at 37 °C. Cells were concentrated by centrifugation (2 min, 140× *g*). According to the established protocol, Vero cells were modified by electroinserting the anti-PSA antibody into their membrane. Briefly, 2.5 × 10^6^ cells in 40 μL PBS were incubated on ice with 400 μL of antibody for 20 min. After incubation the mixture was transferred to an electric field of 1800V/cm and two square electric pulses were applied according to a procedure described previously [20,21] (Figure 1A). Finally, after modification the cells were stored at 37 °C, in 5% CO_2_. The next day cells were counted and experiments were performed with PSA solutions of previously known concentration and blood-serum samples. In a separate set of experiments, cells were electroporated by electroinserting different concentrations of antibody, 0.25 μg/mL, 0.5 μg/mL and 1 μg/mL.

### 2.3. Point-of-Care (POC) System Configuration

The POC system is a custom-made 8× channel potentiostat with dimensions: 11.7 cm × 9.2 cm × 3.4 cm and weight 290 g (Bio-Logic Science Instruments, Seyssinet-Pariset, France). The system recorded the electric signals from cells on the working electrode and allowed rapidity of assay (duration: 5 min). In order to simplify high throughput screening, a strip of eight screen printed electrodes (DRP-8X110, DropSens (Llanera, Asturias, Spain)) were inserted into the potentiostat system (Figure 1B). Each strip of the 8x electrodes pairs composed of a 0.5-mm-thick ceramic substrate with three screen-printed electrodes (WE: carbon, RE: Ag/AgCl, CE: carbon). Hence, eight individual measurements were permitted in parallel.

### 2.4. Assay Procedure

Membrane-engineered cells were microscopically counted with the use of hemocytometer and prepared at density of 1.1 × 10^6^ cells/mL. A drop of 45 μL of cell-containing solution was deposited on each of the eight electrodes and directly another drop of 5 μL of sample was added with the aid of a pipette. The cellular response to the different samples (standard solutions and patient samples) was recorded as a time-series of membrane potential status. Each measurement lasted 300 s at a sampling rate of 2 Hz (Figure 1A).

### 2.5. Immunoradiometric Assay (IRMA) for PSA Detection in Serum

The quantitative determination of total PSA in human serum was based on the two-site IRMA technique that uses mouse monoclonal antibodies against two different (non competitive) epitopes of the PSA molecule (Immunotech S.R.O. Prague, Czech Republic). Samples were tested following system calibration against a series of standard PSA solutions (0, 1, 3, 10, 30 and 100 ng/mL). In order to evaluate the calibration system two positive controlswere used containing 6.47 ± 1.29 and 28.9 ± 5.7 ng/mL PSA, respectively. Both standard and positivecontrol solutions were made of knownPSA concentrationsdiluted in bovine serum albumin buffer with sodium azide (<0.1%). Whole blood samples were centrifuged (10 min, 1300× *g*) and 100 μLof the resulting serum were mixed with 100 μL of tracer solution (labelled with radioiodine, I-125). Samples, control and standard solutions were incubated in tubes layered with the first, non-radioactive, monoclonal antibody, then the second, labelled antibody was added and mixtures were mechanically shaken (300 rpm) for two hours at room temperature. After incubation, the unbound labelled antibody was washed out. The concentration of the total bound labelled antibody was counted with a Gamma counter (PACKARD Cobra Auto-gamma, model C5002, GMI, Troy, MI, USA) for 60 s. The total PSA concentration in the samples was calculated by interpolation in the standard curve [23].

### 2.6. Electrochemical Immunoassay

Τhe gold nanoparticle-modified screen printed carbon electrodes (DRP-110GNP) used for the parallel electrochemical approach incorporated a screen printed three-electrode configuration, which comprised around-shaped AuNP modified carbon working electrode (diameter: 1.6 mm, geometrical area: 0.0196 cm^2^), a carbon counter electrode and a silver pseudo-reference electrode.AuNPs are widely used in electrode modification since they can amplify the detection signal, improve the electron transducer, and reduce the detection limit in electrochemical biosensors [24,25]. In immunoassays they provide more surface area and higher antibody concentrations can be immobilized on the electrode’s surface. In addition, AuNPs have the ability tο enhance electrode conductivity, thus increasing sensitivity, cost-effectivenes, and the ability to facilitate electron transfer by transducing the binding reaction of antigens at antibody immobilized surfaces [26,27,28].

#### Antibody Immobilization on the Electrode’s Surface

Τhe gold nanoparticle-modified carbon electrodes were first covered with a mixture of BSA/EDC/NHS (10 μL) and then incubated 30 min at room temperature in a humid atmosphere [29]. They were then carefully washed dropwise with acetate buffer and dried under N_2_ flow. 10 μL of a 5:2:2 mixture of BSA (5 mg/mL in Acetate buffer pH 5.6), 0.1 M *N*-Hydroxysuccinimide (NHS) and 0.4 M *N*-(3-dimethylaminopropyl)-*N*′-ethylcarbodiimide (EDC) were deposited on each working electrode. Afterwards, 10 μL of 1:1 of EDC–NHS was placed on the electrode surface (30 min at room temperature), washed and dried under N_2_ flow. The antibody solution (100 ng/mL) was then pipetted over the surface (1 h room temperature, humid atmosphere). For the deactivation of the remaining succinimide groups and in order to block unreacted active sites 1 M ethanolamine–HCl was added for 15 min in the dark [30]. The modified electrodes were stored dry overnight at 4 °C. Various dilutions of PSA standards (0, 1, 5 and 10 ng/mL) were applied onto the electrodes surface and incubated for 1 h at room temperature in a humid atmosphere (to prevent evaporation). After rinsing with acetate buffer 10 μL of polyclonal anti-PSA antibody were added and the electrodes were incubated a under humid atmosphere for 1 h. The assay was then completed by adding polyclonal antibody–HRP conjugate solution after a washing step and incubation for 1 h at room temperature. Different secondary antibody concentrations have been tested (1:7500, 1:500, 1:2500 and 1:1000) and the1:1000 dilution was chosen. All measurements were performed by the addition of 0.015% 3,3′,5,5′-tetramethylbenzidine dihydrochloride (TMB) and 0.01% hydrogen peroxide as a substrate for HRP [31]. Measurements were recorded with a potentiostat device (UniscanSensorStat, Bio-Logic Science Instruments, Seyssinet-Pariset, France) and the data were analysed by the UiEChem™ Research Electrochemistry software package for chronoamperometry experiments at −100 mV for 180 s.

### 2.7. Statistical Analysis

A set of four parallel replicates, was recorded for each sample and each experiment was repeated six times. For the statistical analysis one-way ANOVA was performed by using GraphPad Prism (GraphPad Software, La Jolla, CA, USA). The p value of statistical significance was set at *p* < 0.05.

## 3. Results

### 3.1. Biosensor Response against PSA Standard Solution

**Figure 2:**
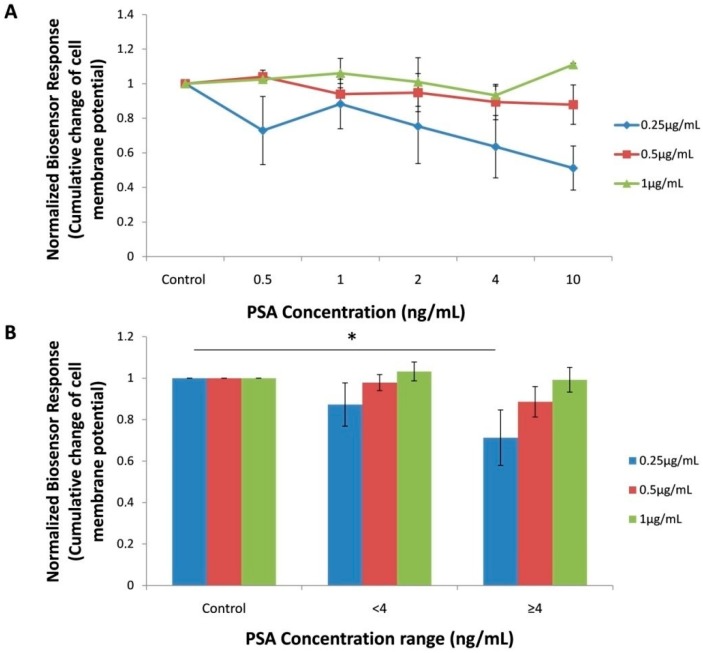
Normalized biosensor response (cumulative change of cell membrane potential) to increasing standard Prostate-specific antigen (PSA) concentrations after electroinserting anti-PSA antibody at different concentrations in membrane-engineered cells. (**A**), Biosensor response to increasing PSA concentration range (**B**) Concentration of the anti-PSA antibody (μg/mL): blue columns 0.25, red columns 0.5, green columns 1. Data are means ± SE of replications (n = 24) received on six different dates using different batches of membrane-engineered cells. * *p* < 0.05, significantly different from control.

Conforming to the working principle of the combined BERA/membrane engineering approach, interaction of a target analyte (presently PSA) with cells engineered with an analyte-specific antibody causes a change in cell membrane potential which is dependent on the concentration of said analyte. In our experiments, membrane-engineered Vero cells with 1 μg/mL anti-PSA antibody, did not perform a significantly different response to different, increasing PSA concentrations. On the contrary, by decreasing the electroinserted anti-PSA antibody concentration to 0.5 μg/mL, a slight decrease of the sensor potential was realizedwith increasing PSA concentrations. Finally, in membrane-engineered cells with 0.25 μg/mL anti-PSA antibody the observed pattern of concentration-dependent decrease of cell membrane potential was more significant (Figure 2A). The fact that the lower anti-PSA antibody concentration (0.25 ng/mL) produced the best results can be explained as follows: in accordance with previous reports [18], increasing the density of electroinserted antibodies on membrane-engineered cells and/or the concentration of target analytes above an upper limit is not associated with a titrimetric between the analyte (PSA) and the membrane- engineered carrier cells with the anti-PSA antibodies. This is due to the fact that the analyte-electroiserted antibody reaction involves an electromechanical stress at the site of antibody area on the membrane, triggering to membrane status changes such as conductivity and porosity [32]. Quite frequently, there is a limitation to the modification of cell membrane potential status caused by the increasing density of electroinserted antibodies, with lower densities producing better resolution of response.

It is well documented that the larger part of men tested for total PSA demonstrate levels under 4 ng/mL. This concentration has generally been used as the cut-off value for association with elevated probability of prostate cancer [33,34,35]. For this reason, the responses of the biosensor against standard PSA solutions were reclassified in three different PSA concentration ranges (0, <4 ng/mL and ≥4 ng/mL) (Figure 2B). Cells membrane-engineered with 0.25 μg/mL anti-PSA antibody demonstrated a considerably higher response against the upper (≥4 ng/mL) PSA concentration range. Therefore, it was decided to use this antibody concentration for manufacturing the membrane-engineered cell biorecognition element for the analysis of the human serum samples, as described in following.

### 3.2. PSA Detection with the Electrochemical Immunoassay

**Figure 3:**
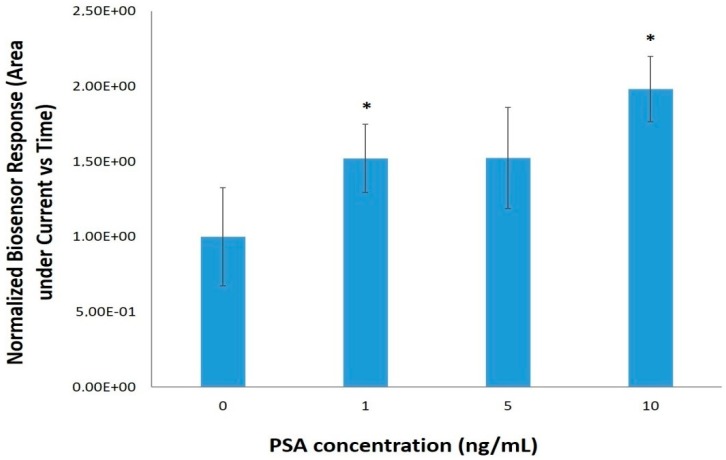
Linear calibration curve of chronoamperometric PSA detection. Plot of normalized immunosensor response of chronoamperograms (area under current vs. time) of HRP/Ab2/Ag/Ab1/BSA/AuNP-C in 0.01% H_2_O_2_ and 0.015% TMB in acetate buffer at pH: 5.6 for various concentrations of PSA (1–10 ng/mL) at an applied potential of −100 mV. * *p* < 0.05, significantly different from control.

Regarding the application of the electrochemical immunoassay for the detection of PSA (2.6) covalent antibody immobilization using bovine serum albumin was carried out in order to enhance the sensitivity of the immunosensor’s device. The main aims for this approach werethe increase of (a) antibody loading and (b) orientation of the antibody binding sites. Different primary antibody concentrations were tested (1, 10, 50, 100 ng/mL) and 100 ng/mL of anti-PSA was chosen as the optimum antibody concentration. Additional tests were performed for the optimization of the secondary HRP conjugated antibody concentration (1:7500, 1:5000, 1:2500, 1:1000) for 10 ng/mL PSA (analytical results not shown). The concentration 1:1000 gave the best signal to noise ration. Figure 3 shows the standard curve for PSA values ranging from 1–10 ng/mL. Chronoamperometry results are expressed as area under current vs. timeagainstPSA concentrations.

The antibody immobilization method used in this approach was amino coupling via activation of the carboxyl groups through NHS and EDC carbodiimide chemistry. Thus, through the NHS esters formation NHS will be eliminated by the reaction with nucleophilic groups of the ligand and a covalent bond will be created. The benefit of this method is that it can be appliedunder relatively mild conditions allowing thus an easy and rapid immobilization. In the present study, this immobilization procedure gave a good sensitivityfor PSA detection in standard solutions especially at concentrations up to 10 ng/mL, where LOD and LOQ values were 1.72 and 9.26 ng/mL PSA, respectively. It should be emphasized, however, that the aim of this study was the development of a rapid system for PSA concentration range above the cut-off value, not the thorough investigation of the biosensor’s analytical limits.

### 3.3. Biosensor Response against Human Serum Samples

**Figure 4:**
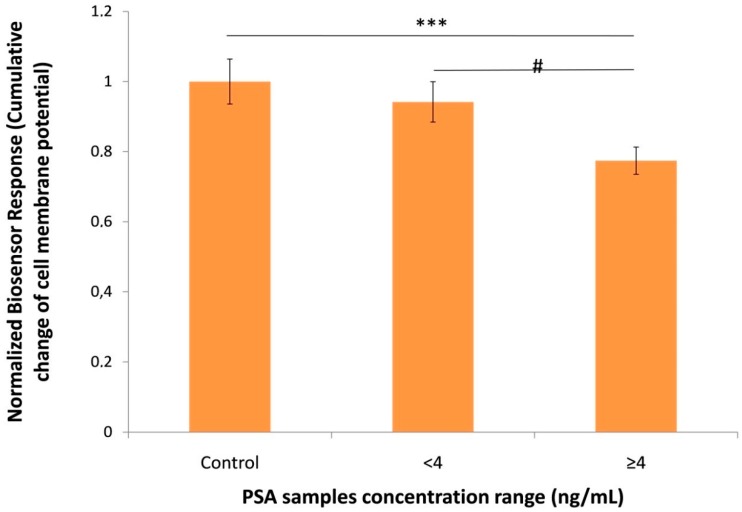
Normalized biosensor response (cumulative change of cell membrane potential) against 39 human serum samplesclassified in three different PSA concentration ranges (0, <4 ng/mL and ≥4 ng/mL). Data are means ± SE of replications (n = 24) received on three different dates using differentbatches of membrane-engineeredcells. *** *p* < 0.001, significantly different from control samples. # *p* < 0.05, significantly different from samples with a PSA concentration <4 ng/mL.

**Figure 5:**
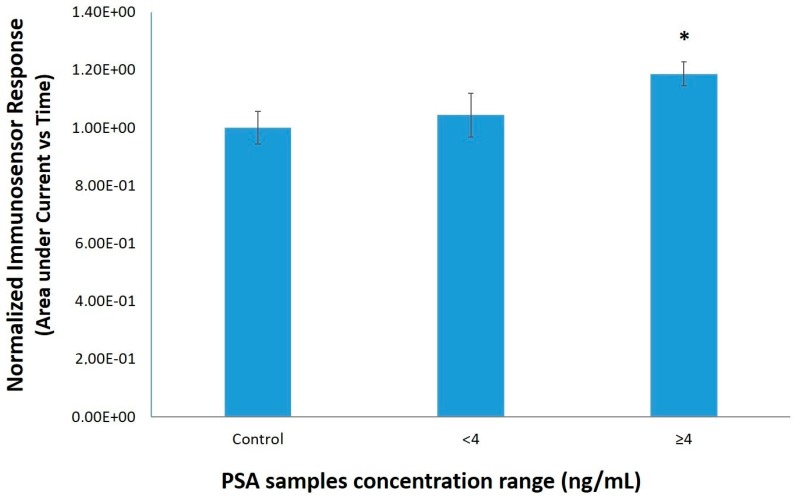
Normalized biosensor response (area under current vs. time) against 39 human serum samples classified in three different PSA concentration ranges (0, <4 ng/mL and ≥4 ng/mL). Data are means ± SE of replications (n = 24) received on three different dates using different batches of immunosensors. * *p* < 0.05, significantly different from control samples with a PSA concentration ≥4 ng/mL.

After the determination of the optimum anti-PSA antibody concentration, the 39 human serum samples were analyzed and the results were shown in Figure 4. As already mentioned under 2.1, the concentration range of the samples by using the immunoradiometric assay was 0–224.4 ng/mL. Similar to standard PSA solutions, the biosensor response against the samples with a PSAconcentration ≥4 ng/mL was significant lower compared to samples with lower concentrations as well as compared to control samples. Similarly, the electrochemical response of the immunosensor against samples with PSA concentrations higher than 4 ng/mL was significantly different from control samples (Figure 5). The cell-based biosensor signal tends to decline as PSA concentration increases whereas the signal of immunosensor follows a reverse pattern. When both methods were compared, the novel cell-based biosensor performed better than the immunosensor in terms of a statistically significantsensitivityand resolution against positive samples containing <4 ng/mL PSA.

## 4. Discussion

In spite of the still controversial association of serum PSA with the early detection of prostate cancer, worldwide PSA screening is steadily increasing as part of routine male health monitoring [36]. The clinical efficiency of PSA screening is certainly increased when combined with digital rectal examination. However, regular testing by the large majority of the population is restricted by the cost of available assays (such as IRMA), calculated at $3822–4956 for year of life saved by prostate cancer screening for men aged 50–70 [37]. It is not surprising, therefore, that the mortality of prostate cancer (though not the incidence) is higher in geographic regions with limited primary health care resources, such as parts of Central and Western Africa [38]. From the perspective of scaling up PSA screening, it may be worth mentioning that the internationalmarket size for prostate cancer diagnostics was estimated at USD 2.2 billion in 2017 and is forecasted to enlarge at a CAGR of 12.3% to reach USD 5.5 billion by 2025 [39]. Therefore, the quest for innovative, high throughput and cost-efficient POC solutions for prostate cancer diagnosis is entirely justified.

However, we should mention that PSA is not a cancer-specific marker and therefore it may give false-positive (increase in PSA levels but no cancer is actually present) or false-negative (low PSA levels even though prostate cancer is not detected) results. Thus, before the final evaluation of every diagnostic tool we must take into account the following considerations:

PSA may be elevatedin malignant as well as enlarged or inflamed prostate conditions such as benign prostate hyperplasia (BPH), prostatitis and others [40,41]. The physiological serum PSA levels range from 0–4 ng/mL and with the development of PCa its serum levels increase [42]. Therefore, PSA is not considered a specific biomarker for PCa detection in the low concentrations between 4–10 ng/mL, since it is quite complicate to provide a clear differencebetween BPH and malignant cases. In addition, a direct correlation between men age and their serum PSAlevel has been found with a PSA increase by about 1 ng/mL every 10 years [43]. Moreover, a recent study suggested that obese men have lower PSA levels, as compared to normal weight men, possibly due to hormonal changes especially by an elevated E2/testosterone ratio and hemodilution [44]. Furthermore, PSA levels are influenced by a number of drugs, such as non-steroidal anti-inflammatory drugs, statins and thiazide diuretics [45,46]. 

Apart from the above mentioned limitations, a significant problem for PSA screening tests is tumor hyper-detection or over-diagnosis, characterized by the detection of a plethora of pathologically insignificant tumors. In a study conducted by the European Prostate Cancer Screening Trial low risk tumors (PSA < 10 ng/mL and Gleason score ≤ 6) were almost three times more common in the screened group than the control group [47,48]. In conjunction with this study, another PIVOT trial that compared the effects of radical prostatectomy versus observation in the PSA era, exhibited there was no benefit from radical surgery for patients with low-risk tumors, which are precisely the majority of cases found in screening programs. Even after 20 years of monitoring for patients with prostate adenocarcinoma with a Gleason score of 6 between there was no difference in mortality those who did and did not undergo surgery [49]. Therefore, for the improvement of sensitivity, enhancement of specificity in PSA low concentrations and for prediction of tumor aggressiveness and PCa morbidity, more indicators such as fPSA/tPSA ratio must also be taken into account [50,51].

In recent years, combined approaches have emerged to provide novel solutions of clinical and commercial value to PSA screening. For example, OPKO Health’s 4Kscore test integrates measured values of four prostate-specific kallikrein proteins (intact PSA, free PSA, total PSA and human Kallikrein-2), also considering the clinical data of the patient in order to calculate a personalized risk for aggressive prostate cancer [52,53]. Another type of test, the Prostate Health Index (PHI) is a mathematical formula that associates free, total and [-2]proPSA into a single score. The test, which is commercially available in the U.S.A., Australia and Europe, has been demonstrated to outperform its individual components screening for the identification of clinically significant prostate cancer [54]. Of interest is also the report by Huo et al. [55], who detected the presence of prostate tumor-associated molecules in blood by applying a dynamic light scattering (DLS) analysis of citrate-protected gold nanoparticles (AuNPs) mixed with human IgG. Following an overnight incubation (4 °C) of the IgG-AuNP solution with spiked blood samples, a change of the average particle size was observed which was in reverse line related to the tumor grade. Consequently, this method is more suitable for determining prostate tumor aggressiveness rather than beingan early diagnostic test, at least in its current version. At the same time, enormous research is being carried out for the identification of novel and clinically efficient blood-based and urinary cancer biomarkers [56,57,58]. In this respect, a number of novel assay concepts have been reported that detect DNA hypermethylation, a characteristic trait of prostate cancer associated with the epigenetic silencing of key genes such as GST-Pi and SOX11 [59,60,61,62].

Taking into consideration the progress described above, we feel that the results of the present study, though preliminary, reflect substantial progress in the development of a clinically relevant biosensor for PSA screening for the following reasons: First of all, the biosensor reported here is based on a combination of the repeatedly proven principles of the Molecular Identification through Membrane Engineering and the Bioelectric Recognition Assay, which have led to the development of commercial screening tests for toxins, viruses and other risk-associated compounds in food safety and medicine [18,19,22]. In other words, the innovative PSA test is founded upon a robust technological concept, and is readily transferable to practical use. Second, the biosensor system is characterized by a true POC nature thanks to its small size (dimensions: 11.7 cm × 9.2 cm × 3.4 cm and weight 290 g), ease of operation, without requirement of reagents and with ultra high speed. A single-step, five-minute-lasting assay was achieved, favourably compared with the multi-step standard IRMA method with an average duration of more than 2.5 h. Third, the innovativesystem was tested against a sufficiently large set of clinical samples and its performance was compared against both standard (IRMA) and other biosensors developed methods for the detection of total PSA. The commercial cost of each assay is estimated below $12 even at a limited scale.

On the other hand, the wide application of the novel biosensor is currently limited by the reduced viability of membrane-engineered cells (not exceeding 2–4 weeks in culture) which dictates the need for a dedicated cell culture facility in the relative vicinity of the PSA diagnostic facilities (diagnostic laboratories, primary healthcare units, hospitals). This limitation could be overcome by using more robust types of cells, such as fish gill cells able to be stored for considerably long periods at room temperature [63]. In addition, the prognostic value of the biosensor would be increased if the system could be expanded to detect free and intact PSA, as well as other prostate-associated protein such as human Kallikrein-2. These additional features lie entirely within the potential of the working principle of the biosensor platform presented in this study. Research is currently in progress to develop these additional options as a proof-of-concept.

## 5. Conclusions

In conclusion, and taking in consideration the merits and disadvantages of the novel approach described above, the cell-based biosensor constructed in the present work could be used as aninitiative for further research for the development of a fast, low-cost, and high-throughput POC screening system for PSA. In combination with other parameters and indicators could be a useful tool for prostate cancer diagnosis. Of course, all considerations mentioned above should be taken into account for further investigation. Future challenges for the generation of a more functional device include decreasing sensor setup time and increasing the shelf life of the biosensor’s assembly.

## References

[1] Fust K. (2015). The Gale Encyclopedia of Cancer.

[2] Hanna L., Crosby T., Macbeth F. (2015). Practical Clinical Oncology.

[3] Ankerst D.P., Tangen C.M., Thompson I.M. (2009). Prostate Cancer Screening.

[4] Makarov D.V., Humphreys E.B., Mangold L.A., Walsh P.C., Partin A.W., Epstein J.I., Freedland S.J. (2006). Pathological outcomes and biochemical progression in men with T1c prostate cancer undergoing radical prostatectomy with prostate specific antigen 2.6 to 4.0 vs. 4.1 to 6.0 ng/mL. J. Urol..

[5] Carter H.B., Pearson J.D., Metter E.J., Brant L.J., Chan D.W., Andres R., Fozard J.L., Walsh P.C. (1992). Longitudinal evaluation of prostate-specific antigen levels in men with and without prostate disease. JAMA.

[6] Vural T., Yaman Y.T., Ozturk S., Abaci S., Denkbas E.B. (2018). Electrochemical immunoassay for detection of prostate specific antigen based on peptide nanotube-gold nanoparticle-polyaniline immobilized pencil graphite electrode. J. Colloid Interface Sci..

[7] Pihikova D., Kasak P., Kubanikova P., Sokol R., Tkac J. (2016). Aberrant sialylation of a prostate-specific antigen: Electrochemical label-free glycoprofiling in prostate cancer serum samples. Anal. Chim. Acta.

[8] Chen H., Huang J., Fam D.W.H., Tok A.I.Y. (2016). Horizontally aligned carbon nanotube based biosensors for protein detection. Bioengineering.

[9] Ji S., Lee M., Kim D. (2018). Detection of early stage prostate cancer by using a simple carbon nanotube@paper biosensor. Biosens. Bioelectron..

[10] Zheng X., Li L., Cui K., Zhang Y., Zhang L., Ge S., Yu J. (2018). Ultrasensitive enzyme-free biosensor by coupling cyclodextrin functionalized au nanoparticles and high-performance au-paper electrode. ACS Appl. Mater. Interfaces.

[11] Yang L., Li N., Wang K., Hai X., Liu J., Dang F. (2018). A novel peptide/Fe_3_O_4_@SiO_2_-Au nanocomposite-based fluorescence biosensor for the highly selective and sensitive detection of prostate-specific antigen. Talanta.

[12] Xu T., Song Y., Gao W., Wu T., Xu L.P., Zhang X., Wang S. (2018). Superwettable electrochemical biosensor toward detection of cancer biomarkers. ACS Sens..

[13] Jolly P., Formisano N., Tkac J., Kasak P., Frost C.G., Estrela P. (2015). Label-free impedimetric aptasensor with antifouling surface chemistry: A prostate specific antigen case study. Sens. Actuators B Chem..

[14] Pihíková D., Belicky Š., Kasák P., Bertok T., Tkac J. (2016). Sensitive detection and glycoprofiling of a prostate specific antigen using impedimetric assays. Analyst.

[15] Spain E., Gilgunn S., Sharma S., Adamson K., Carthy E., O’Kennedy R., Forster R.J. (2016). Detection of prostate specific antigen based on electrocatalytic platinum nanoparticles conjugated to a recombinant scFv antibody. Biosens. Bioelectron..

[16] Yang Z., Kasprzyk-Hordern B., Goggins S., Frost C.G., Estrela P. (2015). A novel immobilization strategy for electrochemical detection of cancer biomarkers: DNA-directed immobilization of aptamer sensors for sensitive detection of prostate specific antigen. Analyst.

[17] Tamboli V.K., Bhalla N., Jolly P., Bowen C.R., Taylor J.T., Bowen J.L., Allender C.J., Estrela P. (2016). Hybrid synthetic receptors on MOSFET devices for detection of prostate specific antigen in human plasma. Anal. Chem..

[18] Moschopoulou G., Vitsa K., Bem F., Vassilakos N., Perdikaris A., Blouhos P., Yialouris C., Frossiniotis D., Anthopoulos I., Maggana O. (2008). Engineering of the membrane of fibroblast cells with virus-specific antibodies: A novel biosensor tool for virus detection. Biosens. Bioelectron..

[19] Perdikaris A., Alexandropoulos N., Kintzios S. (2009). Development of a Novel, Ultra-rapid Biosensor for the Qualitative Detection of Hepatitis B Virus-associated Antigens and Anti-HBV, Based on “Membrane-engineered” Fibroblast Cells with Virus-Specific Antibodies and Antigens. Sensors.

[20] Moschopoulou G., Valero T., Kintzios S. (2012). Superoxide determination using membrane-engineered cells: An example of a novel concept for the construction of cell sensors with customized target recognition properties. Sens. Actuators B Chem..

[21] Apostolou T., Pascual N., Marco M.P., Moschos A., Petropoulos A., Kaltsas G., Kintzios S. (2014). Extraction-less, rapid assay for the direct detection of 2,4,6-trichloroanisole (TCA) in cork samples. Talanta.

[22] Mavrikou S., Flampouri E., Iconomou D., Kintzios S. (2017). Development of a cellular biosensor for the detection of aflatoxin B1, based on the interaction of membrane engineered Vero cells with anti-AFB1 antibodies on the surface of gold nanoparticle screen printed electrodes. Food Control.

[23] Foroutan H., Najafi R., Babaei M.H., Shafii M. (2008). Preparation of prostate specific antigen standards for immunoradiometric assay. Iran. J. Radiat. Res..

[24] Lin Y., Chen S., Chuang Y., Lu Y.C., Shen T.Y., Chang C.A., Lin C.S. (2008). Disposable amperometricimmunosensing strips fabricated by Au nanoparticles-modified screen-printed carbon electrodes for the detection of foodborne pathogen *Escherichia coli* O157: H7. Biosens. Bioelectron..

[25] Chen S.H., Chuang Y.C., Lu Y.C., Lin H.C., Yang Y.L., Lin C.S. (2009). A method of layer-by-layer gold nanoparticles hybridization in a quartz crystal microbalance DNA sensing system used to detect dengue virus. Nanotechnology.

[26] Pingarro’n J.M., Ya’nez-Sedeno P., Araceli G. (2008). Gold-nanopartcle-based electrochemical biosensors. Electrochim. Acta.

[27] Goyal R.N., Gupta V.K., Oyama M., Bachheti N. (2006). Differential pulse voltammetric determination of atenolol in pharmaceutical formulations and urine using nanogold modified indium tin oxide electrode. Electrochem. Commun..

[28] Jacobs M., Jacobs M.J., Selvam A.P., Craven J.E., Prasad S. (2014). Antibody-conjugated gold nanoparticle-based immunosensor for ultra-sensitive detection of troponin-T. J. Lab. Autom..

[29] Polonschii C., David S., Tombelli S., Mascini M., Gheorghiu M. (2010). A novel low-cost and easy to develop functionalization platform. Case study: Aptamer-based detection of thrombin by surface plasmon resonance. Talanta.

[30] Badea M., Floroian L., Restani P., Cobzac S.C.A., Moga M. (2016). Ochratoxin A Detection on Antibody-Immobilized on BSA-Functionalized Gold Electrodes. PLoS ONE.

[31] Kadir M.K., Tothill I.E. (2010). Development of an electrochemical immunosensor for fumonisins detection in foods. Toxins.

[32] Kokla A., Blouchos P., Livaniou E., Zikos C., Kakabakos S.E., Petrou P.S., Kintzios S. (2013). Visualization of the membrane engineering concept: Evidence for the specific orientation of electroinserted antibodies and selective binding of target analytes. J. Mol. Recognit..

[33] Malati T., Kumari G.R. (2004). Racial and ethnic variation of PSA in global population: Age specific reference intervals for serum prostate specific antigen in healthy South Indian males. Indian J. Clin. Biochem..

[34] Luboldt H.J., Schindler J.M., Rubben H. (2007). Age-Specific Reference Ranges for Prostate-Specific Antigen as a Marker for Prostate Cancer. EAU-EBU Updat. Ser..

[35] Erol B., Gulpinar M.T., Bozdogan G., Ozkanli S., Onem K., Mungan G., Bektasf S., Tokgoz H., Akduman B., Mungan A. (2014). The cutoff level of free/total prostate specific antigen (f/t PSA) ratios in the diagnosis of prostate cancer: A validation study on a Turkish patient population in different age categories. Kaohsiung J. Med. Sci..

[36] Shahyad S., Saadat S.H., Hosseini-Zijoud S.M. (2018). The Clinical Efficacy of Prostate Cancer Screening in Worldwide and Iran: Narrative Review. World J. Oncol..

[37] Benoit R.M., Gronberg H., Naslund M.J. (2001). A quantitative analysis of the costs and benefits of prostate cancer screening. Prostate Cancer Prostatic Dis..

[38] Globocan 2018 Graph Production: IARC. http://gco.iarc.fr/today.

[39] Grand View Research 2018. Prostate Cancer Diagnostics Market Analysis Report by Type (Preliminary Tests, Confirmatory Tests), by Region (North America, APAC, Europe, MEA, Latin America), And Segment Forecasts, 2018–2025. https://www.grandviewresearch.com/industry-analysis/prostate-cancer-diagnostics-market.

[40] Malati T., Kumari G.R., Murthy P.V., Reddy C.R., Prakash B.S. (2006). Prostate specific antigen in patients of benign prostate hypertrophy and carcinoma prostate. Indian J. Clin. Biochem..

[41] Yamamoto M., Hibi H., Miyake K. (1993). Prostate-specific antigen levels in acute and chronic bacterial prostatitis. Hinyokika Kiyo.

[42] Stamey T.A., Yang N., Hay A.R., McNeal J.E., Freiha F.S., Redwine E. (1987). Prostate-specific antigen as a serum marker for adenocarcinomaof the prostate. N. Engl. J. Med..

[43] Oesterling J.E., Jacobsen S.J., Chute C.G., Guess H.A., Girman C.J., Panser L.A., Lieber M.M. (1993). Serum prostate-specific antigenin a community-based population of healthy men. Establishmentof age-specific reference ranges. JAMA.

[44] Aref A.T., Vincent A.D., O’Callaghan M.E., Martin S.A., Sutherland P.D., Hoy A.J., Butler L.M., Wittert G.A. (2018). The inverse relationship between prostate specific antigen (PSA) and obesity. Endocr. Relat. Cancer.

[45] Iguchi K., Hashimoto M., Kubota M., Yamashita S., Nakamura M., Usui S., Sugiyama T., Hirano K. (2014). Effects of 14 frequently used drugs on prostate-specific antigen expression in prostate cancer LNCaP cells. Oncol. Lett..

[46] Chang S.L., Harshman L.C., Presti J.C. (2010). Impact of common medications on serum total prostate-specific antigen levels: Analysis of the National Health and Nutrition Examination Survey. J. Clin. Oncol..

[47] Schröder F.H., Hugosson J., Roobol M.J., Tammela T.L., Ciatto S., Nelen V., Kwiatkowski M., Lujan M., Lilja H., Zappa M. (2012). ERSPC Investigators. Prostate-cancer mortality at 11 years of follow-up. N. Engl. J. Med..

[48] Roobol M.J., Kranse R., Bangma C.H., van Leenders A.G., Blijenberg B.G., van Schaik R.H., Kirkels W.J., Otto S.J., van der Kwast T.H., de Koning H.J. (2013). ERSPC Rotterdam Study Group. Screening for prostate cancer: Results of the Rotterdam section of the European randomized study of screening for prostate cancer. Eur. Urol..

[49] Wilt T.J., Jones K.M., Barry M.J., Andriole G.L., Culkin D., Wheeler T., Aronson W.J., Brawer M.K. (2017). Follow-up of prostatectomy versus observation for early prostate cancer. N. Engl. J. Med..

[50] Ito K., Yamamoto T., Ohi M., Kurokawa K., Suzuki K., Yamanaka H. (2003). Free/total PSA ratio is a powerful predictor of future prostatecancer morbidity in men with initial PSA levels of 4.1 to 10.0ng/mL. Urology.

[51] Catalona W.J., Partin A.W., Slawin K.M., Brawer M.K., Flanigan R.C., Patel A., Richie J.P., deKernion J.B., Walsh P.C., Scardino P.T. (1998). Use of the percentage of free prostatespecificantigen to enhance differentiation of prostate cancer frombenign prostatic disease: A prospective multicenter clinical trial. JAMA.

[52] Borque-Fernando A., Rubio-Briones J., Esteban L.M., Dong Y., Calatrava A., Gómez-Ferrer Á., Gómez-Gómez E., Gil Fabra J.M., Rodríguez-García N., López González P.Á. (2018). Role of the 4Kscore test as a predictor of reclassification in prostate cancer active surveillance. Prostate Cancer Prostatic Dis..

[53] Vickers A.J., Vertosick E.A., Sjoberg D.D. (2018). Value of a Statistical Model Based on Four Kallikrein Markers in Blood, Commercially Available as 4Kscore, in All Reasonable Prostate Biopsy Subgroups. Eur. Urol..

[54] Loeb S., Sanda M.G., Broyles D.L., Shin S.S., Bangma C.H., Wei J.T., Partin A.W., Klee G.G., Slawin K.M., Marks L.S. (2015). The Prostate Health Index Selectively Identifies Clinically Significant Prostate Cancer. J. Urol..

[55] Huo Q., Litherland S.A., Sullivan S., Hallquist H., Decker D.A., Rivera-Ramirez I. (2012). Developing a nanoparticle test for prostate cancer scoring. J. Transl. Med..

[56] Anceschi U., Tuderti G., Lugnani F., Biava P., Malossini G., Luciani L., Cai T., Marsiliani D., Filianoti A., Mattevi D. (2018). Novel diagnostic biomarkers of prostate cancer: An update. Curr. Med. Chem..

[57] Hendriks R.J., van Oort I.M., Schalken J.A. (2017). Blood-based and urinary prostate cancer biomarkers: A review and comparison of novel biomarkers for detection and treatment decisions. Prostate Cancer Prostatic Dis..

[58] Gadzinski A.J., Cooperberg M.R. (2018). Prostate Cancer Markers. Cancer Treat. Res..

[59] Pugongchai A., Bychkov A., Sampatanukul P. (2017). Promoter hypermethylation of SOX11 correlates with adverse clinicopathological features of human prostate cancer. Int. J. Exp. Pathol..

[60] Ashour N., Angulo J.C., Andrés G., Alelú R., González-Corpas A., Toledo M.V., Rodríguez-Barbero J.M., López J.I., Sánchez-Chapado M., Ropero S. (2014). A DNA hypermethylation profile reveals new potential biomarkers for prostate cancer diagnosis and prognosis. Prostate.

[61] Aref-Eshghi E., Schenkel L.C., Ainsworth P., Lin H., Rodenhiser D.I., Cutz J.C., Sadikovic B. (2018). Genomic DNA Methylation-Derived Algorithm Enables Accurate Detection of Malignant Prostate Tissues. Front. Oncol..

[62] Baden J., Adams S., Astacio T., Jones J., Markiewicz J., Painter J., Trust C., Wang Y., Green G. (2011). Predicting prostate biopsy result in men with prostate specific antigen 2.0 to 10.0 ng/mL using an investigational prostate cancer methylation assay. J. Urol..

[63] Maunder R.J., Baron M.G., Owen S.F., Jha A.N. (2017). Investigations to extend viability of a rainbow trout primary gill cell culture. Ecotoxicology.

